# COVID-19 Pandemic–Related Changes in Rates of Neonatal Abstinence Syndrome

**DOI:** 10.1001/jamanetworkopen.2024.1651

**Published:** 2024-03-08

**Authors:** Sarka Lisonkova, Jeffrey N. Bone, Qi Wen, Giulia M. Muraca, Joseph Y. Ting, Neda Razaz, K. S. Joseph

**Affiliations:** 1 Department of Obstetrics and Gynaecology, University of British Columbia and the Children’s and Women’s Hospital and Health Centre of British Columbia, Vancouver, British Columbia, Canada; 2 School of Population and Public Health, University of British Columbia, Vancouver, British Columbia, Canada; 3 Division of Biostatistics, British Columbia Children’s Hospital Research Institute, Vancouver, British Columbia, Canada; 4 Department of Health Research Methods, Evidence and Impact, McMaster University, Hamilton, Ontario, Canada; 5 Department of Obstetrics and Gynecology, McMaster University, Hamilton, Ontario, Canada; 6 Department of Pediatrics, University of Alberta, Edmonton, Alberta, Canada; 7 Clinical Epidemiology Division, Department of Medicine, Karolinska Institutet, Stockholm, Sweden

## Abstract

This cross-sectional study examines COVID-19 pandemic–related changes in rates of neonatal abstinence syndrome (NAS) and whether infants in urban or rural areas and those with low socioeconomic status were disproportionately affected.

## Introduction

Although opioid overdose deaths increased during the COVID-19 pandemic,^1^ changes in rates of neonatal abstinence syndrome (NAS) have not been adequately studied. We examined pandemic-related changes in rates of NAS and whether infants in urban vs rural areas and those with low socioeconomic status (SES) were disproportionately affected.

## Methods

This cross-sectional study included all live births at 20 weeks’ or more gestation in British Columbia (BC), Canada, between 2010 to 2011 and 2021 to 2022, with data obtained from the BC Perinatal Database Registry.^2^ NAS cases were identified using *International Statistical Classification of Diseases and Related Health Problems, Tenth Revision*, diagnostic code P96.1 (eMethods in Supplement 1). Rural residence was defined as residence in a community with fewer than 10 000 inhabitants,^3^ and low or high SES was defined as maternal residence in the lowest or highest neighborhood income quintile, respectively. Ethics approval was obtained from the University of British Columbia, which waived consent because deidentified data were used. The study followed the STROBE reporting guideline.

An interrupted time-series approach, with segmented Poisson regression, was used to assess changes in NAS frequency and temporal trends. The unit of analysis was the month, and the interruption was set to June 1, 2020, to identify infant effects after at least 2 to 3 months of in utero opioid exposure after the March 2020 pandemic onset. Secondary analyses assessed rural vs urban and SES differences in pandemic-related changes in NAS. We further compared prepandemic vs pandemic trends in preterm birth (<37 weeks’ gestation), birth weight, and length of hospital stay (LOS) among infants with NAS. Statistical analysis was performed with R software; all *P* values were from 2-sided tests, and results were deemed statistically significant at *P* < .05.

## Results

The study included 514 189 live-born infants; 2165 had NAS (4.2 per 1000 live births). Between fiscal years 2010 to 2011 (April 1, 2010, to March 31, 2011) and 2019 to 2020 (April 1, 2019, to March 31, 2020; prepandemic period), NAS increased from 2.6 to 4.8 per 1000 live births (Table). Rates were highest in fiscal year 2020 to 2021 and decreased in 2021 to 2022 (5.6 and 4.7 per 1000 live births, respectively). The direction of the temporal trend in NAS rates changed after the pandemic onset in June 2020 (Figure). The relative increase in the NAS rate during the prepandemic period (March 2010 to May 2020) was 0.5% (95% CI, 0.4%-0.6%) per month, and the NAS rate decreased by 1.3% (95% CI, 0.3%-2.6%) per month during the pandemic period (June 2020 to March 2022). There were no large rural vs urban and SES differences or pandemic-related changes in preterm birth, birth weight, and LOS among infants with NAS (Table).

**Table.** Numbers and Rates of NAS Cases Among Infants in British Columbia, Canada, Between 2010-2011 and 2021-2022

Fiscal year	All infants			Infants in urban areas			Infants with low SES			Infants with NAS		
	Live births, No.	NAS cases, No.	Rate per 1000	Live births, No.	NAS cases, No.	Rate per 1000	Live births	NAS cases, No.	Rate per 1000	PTBs, No. (%)	Birth weight, mean (SD), g	LOS, median (IQR), d
2010-2011	42 895	110	2.6	38 912	97	2.5	8750	36	4.1	28 (25.5)	3006 (631)	17 (5-31)
2011-2012	43 039	155	3.6	38 982	138	3.5	8860	72	8.1	36 (23.2)	3005 (606)	18 (6-31)
2012-2013	43 278	141	3.3	39 413	121	3.1	8884	48	5.4	30 (21.3)	2970 (645)	17 (6-33)
2013-2014	42 529	153	3.6	38 597	128	3.3	8496	55	6.5	36 (23.5)	2960 (536)	12 (4-28)
2014-2015	43 131	161	3.7	39 661	141	3.6	6387	50	7.8	38 (23.6)	3067 (538)	11 (5-29)
2015-2016	43 157	206	4.8	39 919	186	4.7	6414	48	7.5	45 (21.8)	2925 (617)	17 (6-30)
2016-2017	43 913	205	4.7	40 508	183	4.5	6431	65	10.1	55 (26.8)	2928 (606)	16 (5-29)
2017-2018	43 407	221	5.1	39 895	193	4.8	6344	70	11.0	71 (32.1)	3009 (646)	18 (5-31)
2018-2019	42 513	179	4.2	38 923	157	4.0	6218	45	7.2	48 (26.8)	2955 (616)	19 (6-29)
2019-2020	42 653	205	4.8	38 610	176	4.6	6071	53	8.7	64 (31.2)	2971 (547)	17 (5-31)
2020-2021	41 088	229	5.6	38 218	198	5.2	5978	77	12.9	76 (33.2)	2944 (617)	17 (5-36)
2021-2022	42 586	200	4.7	39 586	170	4.3	5984	52	8.7	59 (29.5)	2910 (511)	20 (7-34)

Abbreviations: LOS, length of hospital stay; NAS, neonatal abstinence syndrome; PTBs, preterm births; SES, socioeconomic status.

**Figure.**
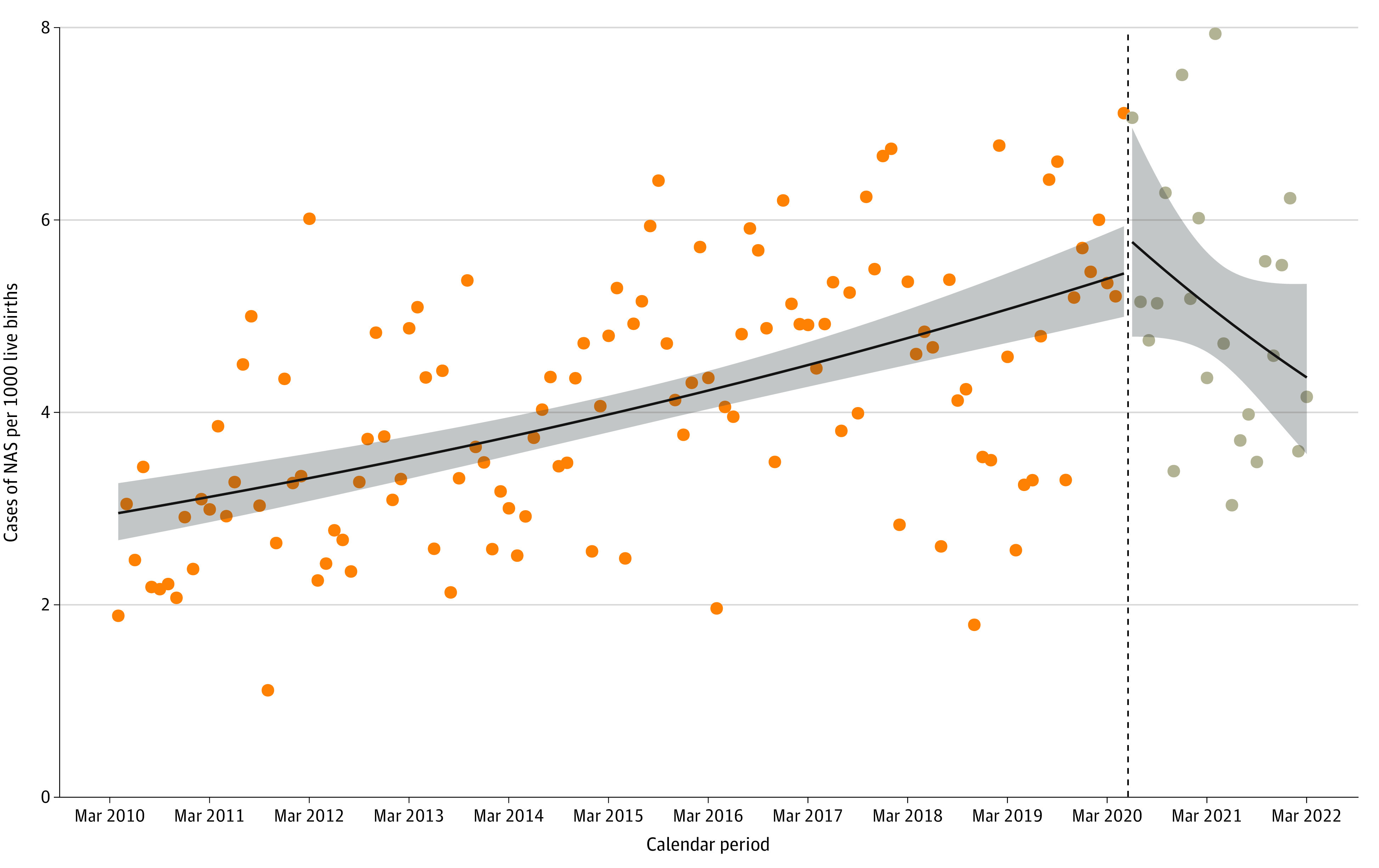
COVID-19 Pandemic–Related Changes in the Incidence of Neonatal Abstinence Syndrome (NAS) in British Columbia, Canada, March 2010 to March 2022 Interrupted time series model with month of each year as the unit of analysis. The interruption was set to June 1, 2020 (dashed vertical line), to identify infants with NAS who would have been born after 2 to 3 months of in utero exposure to opioids after the onset of the COVID-19 pandemic in March 2020. The shaded area indicates the 95% CI.

## Discussion

The rate of NAS in BC, Canada, increased during the prepandemic period and then decreased in the first 2 years of the pandemic. These trends were relatively uniform across rural and urban areas and SES levels. There were no pandemic-related changes in preterm birth, birth weight, and LOS among infants with NAS, suggesting that NAS severity and associated morbidity were unchanged.

Our results are unexpected because studies have documented increased opioid use during the pandemic.^1,4,5^ The continued increase in opioid-related mortality among males and females in BC^6^ was not matched by a continued increase in the incidence of NAS, which decreased in 2021 and 2022. Possible explanations include differences in substance use behavior among pregnant women vs other adults and increases in stillbirths and early neonatal deaths among these women (both preclude a diagnosis of NAS). Changes in fertility rates among women using opioids or improved access to health services after the initial pandemic-related restrictions may have also been associated with the decrease in NAS rates. Lack of details on specific drugs, including treatments (eg, buprenorphine or methadone) used during pregnancy, and regarding stillbirths among women with substance use are limitations of our study.

The increasing prepandemic trend in the rate of NAS was reversed during the pandemic in BC. Future research is needed to corroborate these findings and to address details regarding pandemic-related changes in opioid use and opioid addiction treatments during pregnancy.

## Notes

Supplement 1.
eMethods.

eReference.

Supplement 2.
Data Sharing Statement
