# Supplementary material for: COVID-19 Pandemic–Related Changes in Rates of Neonatal Abstinence Syndrome
Source: JAMA Netw Open. 2024 Mar 8;7(3):e241651. doi: 10.1001/jamanetworkopen.2024.1651 (PMC10924237; doi:10.1001/jamanetworkopen.2024.1651)
Supplement: Supplement 1. — eMethods. eReference. [file jamanetwopen-e241651-s001.pdf]

Supplementary Online Content

Lisonkova S, Bone JN, Wen Q, et al. COVID-19 pandemic–related changes in rates of neonatal abstinence syndrome. *JAMA Netw Open.* 2024;7(3):e241651. doi:10.1001/jamanetworkopen.2024.1651

eMethods.

eReference.

This supplementary material has been provided by the authors to give readers additional information about their work.

© 2024 Lisonkova S et al. *JAMA Network Open.*

eMethods.

NAS was identified by ICD-10-CA code P96.1 (“Neonatal withdrawal symptoms from maternal use of drugs of addiction”) for neonatal abstinence syndrome during newborn hospitalization. This code is different from the code P96.2 (“Withdrawal symptoms from therapeutic use of drugs in newborn”), which is used for neonatal withdrawal syndrome due to therapeutic drugs used to treat neonatal conditions. The ICD-10 code for NAS (P96.1) has shown a high accuracy in a large validation study in the United States using hospital administrative data with a positive predictive value of 98.2%.[1]

© 2024 Lisonkova S et al. *JAMA Network Open.*

eReference.

1. Maalouf FI, Cooper WO, Stratton SM, Dudley JA, Ko J, Banerji A, Patrick SW. Positive Predictive Value of Administrative Data for Neonatal Abstinence Syndrome. Pediatrics. 2019 Jan;143(1):e20174183. doi: 10.1542/peds.2017-4183. Epub 2018 Dec 4. PMID: 30514781; PMCID: PMC6317565.

© 2024 Lisonkova S et al. *JAMA Network Open.*
