# Supplementary material for: COVID-19 Pandemic–Related Changes in Rates of Neonatal Abstinence Syndrome
Source: JAMA Netw Open. 2024 Mar 8;7(3):e241651. doi: 10.1001/jamanetworkopen.2024.1651 (PMC10924237; doi:10.1001/jamanetworkopen.2024.1651)
Supplement: Supplement 2. — Data Sharing Statement [file jamanetwopen-e241651-s002.pdf]

# Data Sharing Statement

Lisonkova. COVID-19 Pandemic–Related Changes in Rates of Neonatal Abstinence Syndrome. *JAMA Netw Open*. Published March 08, 2024. doi:10.1001/jamanetworkopen.2024.1651

**Data**
**Data available:** No

**Additional Information**
**Explanation for why data not available:** Data are available from Population Data BC, Canada, upon request.
